# Characteristics of U.S. adults taking prescription antipsychotic medications, National Health and Nutrition Examination Survey 2013–2018

**DOI:** 10.1186/s12888-020-02895-4

**Published:** 2020-10-01

**Authors:** Jeff A. Dennis, Lisaann S. Gittner, J. Drew Payne, Kenneth Nugent

**Affiliations:** 1 Department of Public Health, Texas Tech University Health Sciences Center, 3601 4th St., MS 9430, Lubbock, TX 79430 USA; 2 Department of Internal Medicine, Texas Tech University Health Sciences Center, Lubbock, TX USA

**Keywords:** Antipsychotic medications, Epidemiology, Chronic disease comorbidities, Antipsychotic medication prevalence

## Abstract

**Background:**

Global 12-month psychosis prevalence is estimated at roughly 0.4%, although prevalence of antipsychotic use in the U.S. is estimated at roughly 1.7%. Antipsychotics are frequently prescribed for off label uses, but have also been shown to carry risk factors for certain comorbid conditions and with other prescription medications. The study aims to describe the socio-demographic and health characteristics of U.S. adults taking prescription antipsychotic medications, and to better understand the association of antipsychotic medications and comorbid chronic diseases.

**Methods:**

The study pools 2013–2018 data from the National Health and Nutrition Examination Survey (NHANES), a nationally representative cross-sectional survey of non-institutionalized U.S. residents (*n* = 17,691). Survey staff record prescription medications taken within the past 30 days for each respondent, from which typical and atypical antipsychotic medications were identified.

**Results:**

Prevalence of antipsychotic use among U.S. adults was 1.6% (*n* = 320). Over 90% of individuals taking antipsychotics reported having health insurance and a usual place for care, significantly more than their counterparts not taking antipsychotics. Further, those taking antipsychotics reported higher prevalence of comorbid chronic diseases and took an average of 2.3 prescription medications more than individuals not taking antipsychotics. Individuals taking antipsychotics were more likely to sleep 9 or more hours per night, be a current smoker, and have a body mass index greater than 30 kg/m^2^.

**Conclusions:**

U.S. adults who take antipsychotic medications report more consistent health care access and higher prevalence of comorbid chronic diseases compared to those not taking antipsychotics. The higher comorbidity prevalence and number of total prescriptions highlight the need for careful assessment and monitoring of existing comorbidities and potential drug-drug interactions among adults taking antipsychotics in the U.S.

## Background

National estimates of United States (U.S.) serious mental illness prevalence vary widely because clear diagnosis requires an in-depth clinical assessment, a process not ideally suited for data collection methods of most large national surveys [1]. Recent meta-analysis suggests global 12-month psychosis prevalence of 0.4% and global lifetime psychosis prevalence of 0.7% [2]. U.S. depression prevalence has been estimated at approximately 20% using validated self-report questionnaires, but medication use is also relevant, as the same study found over half of the 10% of U.S. adults taking antidepressants reported no depressive symptoms [3].

Over two-thirds of adults with a mental disorder report at least one other comorbid health diagnosis, suggesting treatment of mental illness frequently requires careful consideration of concurrent pharmaceutical treatments [4]. Recent commentary suggests comprehensive understanding of the effects of drug-drug interactions with antipsychotics is deficient [5]. Antipsychotics have been shown to have adverse effects on individuals with metabolic syndrome, and may require careful monitoring in individuals with co-morbid disease [6, 7]. A study of Medicaid claims data suggested as many as 25% of individuals taking antipsychotics for schizophrenia were also taking another prescription drug with a potentially harmful interaction [8]. Further, antipsychotics may be used for off-label treatments, and controlled trials have found benefits of atypical antipsychotics to treat dementia, insomnia, obsessive-compulsive disorder, generalized anxiety disorder and other conditions [9, 10].

A study of U.S. outpatient data found antipsychotic medications prescribed in 1.7% of adult office visits, increasingly from non-psychiatrist physicians [11]. National outpatient data provide useful information on prevalence of antipsychotic use, diagnostic categories, and insurance type, but far less information on health care access, health behaviors, and concurrent disease comorbidities. The study has two primary aims: 1) to describe the socio-demographic, health care access, and health behavior characteristics of U.S. adults taking prescription antipsychotic medications; and 2) to compare comorbid diagnoses in individuals taking and not taking antipsychotic medications by exploring which factors explain differences.

## Methods

The National Health and Nutrition Examination Survey (NHANES) is a biennial nationally representative sample of non-institutionalized individuals in the U.S. [12] Respondents age 18 and older from the 2013–18 NHANES were pooled to produce a larger sample size of individuals taking antipsychotic medications (*n* = 320). The 2013–2018 NHANES data includes 17,961 individuals age 18 and up.

A medication inventory is conducted by NHANES interviewers, who ask survey respondents to show any prescriptions with information entered directly from the medication bottle they have taken in the past 30 days [2]. Antipsychotic medications were identified through an inventory of all medications listed in NHANES 2013–18, and included seven typical (chlorpromazine, fluphenazine, haloperidol, loxapine, perphenazine, prochlorperazine, trifluoperazine) and twelve atypical (aripiprazole, asenapine, brexpiprazole, cariprazine, clozapine, iloperidone, lurasidone, olanzapine, paliperidone, quetiapine, risperidone, ziprasidone) antipsychotic medications. Analyses combine typical and atypical antipsychotics because typical antipsychotics were prescribed too infrequently for separate analysis (11.0%, *n* = 42). Approximately 91% (*n* = 286) of respondents reported taking atypical antipsychotics (a small number reported taking both typical and antitypical antipsychotics, but not enough for meaningful population-level analysis).

Standard demographic characteristics include age groups, sex, race/ethnicity, and education level. Health care access and use are measured using questions on insurance status, recent hospitalization, having a usual place for health care, and recent visit to see a mental health professional. A comorbidity score was created based on self-reports of six conditions, including diabetes, hypertension, high cholesterol, cancer, chronic obstructive pulmonary disease (COPD), and coronary heart disease. Comorbidity scores range from 0 to 6. Health variables also include self-reported health, typical hours of sleep on weekdays, and smoking status. Body mass index (BMI) is calculated based on height and weight measurements recorded by NHANES staff.

Respondents self-reported up to three reasons for medication recorded. Although ideally these reasons could be carefully defined as off label vs. indicated use, such a determination was not possible with the information provided, which included a number of reasons listed as “unspecified” types of mental illness. As such, we more conservatively report aggregate frequencies of the different reasons given for taking each medication. As a national sample, NHANES is not intended for reporting individual cases or rare responses. Therefore, we aggregate the reasons for antipsychotic medication use into categories to meet the NHANES minimum reliability sample size threshold of 30 [11, 12].

Analyses use population weighting in Stata 15.1 to account for NHANES’ complex survey design. Chi-square tests are used to compare differences between antipsychotic medication status and categorical demographic and health variables. Wald tests are used to compare mean differences in continuous variables. Ordinary least squares (OLS) regression is used to predict characteristics associated with the number of comorbidities. NHANES is de-identified, publicly available data and therefore the study did not require institutional review board approval.

## Results

Table 1 shows 1.6% of adults report taking antipsychotic medications, the equivalent of approximately 3.8 million U.S. adults during the time frame. Individuals taking antipsychotic medications were more often between the ages of 45–64 and had lower levels of education than individuals not taking antipsychotic medications. About 23% of individuals taking antipsychotic medications report a hospitalization in the past 12 months compared to roughly 10% in the rest of the population. Over 90% of those taking antipsychotics report having health insurance and a usual place for health care, although only about 76% report having seen a mental health professional in the past year.

**Table 1:** Demographics and health care access of U.S. adult population taking antipsychotic medications, NHANES 2013–2018

	Not taking antipsychotic medication	Taking antipsychotic medication		
	N (Weighted %)	N (Weighted %)	chi-square	*p*-value
Overall	17,641 (98. 4)	320 (1.6)		
Female	9151 (51.8)	167 (53.3)	0.2512	0.7014
Male	8490 (48.2)	153 (46.7)		
Age				
18–44	7820 (46.6)	109 (37.2)	33.5398	< 0.001
45–64	5693 (34.1)	155 (50. 4)		
65 plus	4,128 (19.3)	56 (12.5)		
Education				
Less than HS diploma	3631 (13.1)	96 (24.0)	48.7969	< 0.001
HS diploma	3781 (22.5)	83 (27.6)		
Some college	5147 (30.9)	93 (30.6)		
College or above	4,162 (30.3)	39 (16.5)		
Education missing	920 (3.3)	9 (1. 4)		
Race/ethnicity				
Non-Hispanic white	6372 (63.6)	145 (64.8)	7.9594	0.093
Non-Hispanic black	3783 (11. 4)	79 (14.6)		
Hispanic	4,530 (15.6)	55 (10.3)		
Other race	2956 (9. 4)	41 (10. 4)		
Insurance				
Not insured	3249 (15. 4)	24 (7.6)	13.0459	< 0.001
Insured	14,392 (84.6)	296 (92. 4)		
Hospitalized in the past 12 months?				
No	15,649 (89.8)	241 (77. 4)	45.8054	< 0.001
Yes	1984 (10.2)	79 (22.6)		
Have a usual place for health care?				
No	3209 (17.5)	23 (6.8)	22.1397	< 0.001
Yes	14,432 (82.5)	297 (93.2)		
See a mental health professional past 12 months?				
No	16,234 (91.3)	80 (24.0)	1421.8225	< 0.001
Yes	1399 (8.8)	240 (76.0)		

Table 2 shows health characteristics of individuals taking antipsychotic medications. Individuals taking antipsychotic medications reported about 0.3 more comorbid conditions and 2.3 more prescription medications, on average, than individuals not taking antipsychotic medications. Individuals taking antipsychotics reported worse health, were more often obese, more likely to smoke, and slept a longer number of hours than individuals not taking antipsychotics.

**Table 2:** Health characteristics of U.S. adult population taking antipsychotic medications, NHANES 2013–2018

	Not taking antipsychotic medication	Taking antipsychotic medication		
	Mean (SD)	Mean (SD)	F	*p*-value
Comorbidity score^a^	1.0 (1.1)	1.3 (1.3)	20.07^b^	0.001
Number of curent total prescriptions	3.5 (2.9)	5.8 (3.8)	77.02	< 0.001
	N (Weighted %)	N (Weighted %)	chi-square	*p*-value
Self-reported health				
Fair/Poor	4,227 (18.1)	146 (42.6)	133.4917	< 0.001
Good	6525 (35.3)	119 (37. 4)		
Very good/Excellent	6867 (46.7)	55 (20.0)		
Body Mass Index				
Underweight	309 (1.7)	4 (0.8)	12.4518	0.0122
Normal	4,607 (27.5)	74 (23. 4)		
Overweight	5282 (31.6)	73 (26.1)		
Obese	6447 (39.2)	147 (49.7)		
Hours of sleep				
< 7 h	4,987 (30. 4)	63 (20.0)	126.5587	< 0.001
7–8 h	7271 (50.2)	109 (33.0)		
9h hours	3281 (19. 4)	125 (47.0)		
Smoking status				
Never	10,473 (58.0)	115 (38. 4)	78.1164	< 0.001
Former	3934 (23.8)	73 (23.5)		
Current	3221 (18.2)	132 (38.1)		
Source: NHANES 2013–18				

^a^Count of self reported doctor diagnosis of diabetes, hypertension, high cholesterol, COPD, coronary heart disease, cancer

^b^Wald test on comorbidity score and number of medications

Table 3 aggregates reported uses to meet minimum the NHANES sample size threshold of 30 [11, 12]. Approximately one-third of all respondents taking antipsychotics reported major depressive disorder as a reason for use. About 26% reported bipolar disorder and 10% reported schizophrenia as reasons for use. In total, about 62% of respondents taking antipsychotics mentioned at least one of: schizophrenia, bipolar disorder, or major depressive disorder, as a reason for use (results not shown). Insomnia or other sleep disorders were the only off-label reason listed for use that met the minimum sample size threshold, and were reported by about 17% of all persons taking antipsychotics. Over 20% of respondents listed unspecified mental illness as a reason for antipsychotic use, as well as a few approved, but less common uses, such as prochlorperazine for nausea and vomiting [13]. Use of antipsychotics for obsessive compulsive disorder, attention deficit hyperactivity disorder, panic disorder, post-traumatic stress disorder, and degenerative diseases of the nervous system were reported in a small number of cases, but did not meet the sample size threshold of 30, respectively.

**Table 3:** Frequency and percentage of self-reported reasons for antipsychotic medication use, NHANES 2013–2018

Reason listed^a^	N (%)
Schizophrenia	41 (9.6)
Bipolar disorder, unspecified	74 (25.5)
Major depressive disorder, single episode or recurrent	100 (32.3)
Insomnia or other sleep disorder	53 (16.6)
Unspecified psychosis, mood, or anxiety disorder	72 (22.2)

^a^Does not sum to 100% - respondents can report multiple reasons for use

Individuals using antipsychotic medications had a 0.3 higher comorbidity score than individuals who do not take antipsychotics adjusted for age, sex, and race/ethnicity (Table 4). Adjusting for health care access and smoking status reduced the comorbidity in individuals taking antipsychotics association by about 20%, although the relationship remained statistically significant.

**Table 4:** OLS regression predicting comorbidity score, adjusting for demographics and health characteristics, (*N* = 17,961)

	Model 1			Model 2		
	b	95% CI	*p*-value	b	95% CI	*p*-value
Taking antipsychotic medication	0.32	(0.17,0.48)	< 0.001	0.26	(0.10,0.41)	0.002
Age	0.04	(0.04,0.04)	< 0.001	0.03	(0.03,0.04)	< 0.001
Female	−0.11	(−0.15,-0.07)	< 0.001	−0.11	(− 0.16,− 0.07)	< 0.001
Race/ethnicity (NH white = ref)						
NH black	0.05	(0.00,0.09)	0.036	0.07	(0.03,0.12)	0.001
Hispanic	-0.07	(−0.11,-0.02)	0.006	0.01	(−0.04,0.05)	0.706
Other race	−0.05	(−0.12,0.02)	0.141	− 0.02	(− 0.09,0.05)	0.514
Have a usual place for health care				0.24	(0.20,0.29)	< 0.001
Insured				0.14	(0.08,0.19)	< 0.001
Smoking (Never = ref)						
Former smoker				0.19	(0.15,0.24)	< 0.001
Current smoker				0.21	(0.16,0.25)	< 0.001
r-squared	0.323			0.339		

## Discussion

Adult prevalence of antipsychotic use was 1.6%, resembling a recent U.S. outpatient sample estimate of 1.7% [11]. Individuals taking antipsychotic medications reported better overall access to health care, as they were more likely to have health insurance, have seen a mental health professional in the last 12 months, and have a usual place for health care. Lack of insurance or of a usual health care provider may be a barrier to receiving a prescription for antipsychotic medication, or those taking the medications may have incentive to maintain access to keep a consistent prescription. Since individuals taking antipsychotics also report higher prevalence of comorbid chronic diseases, this population may also more frequently interact with health providers for maintenance of these diseases as well. Although the comorbidity score is not a comprehensive list of conditions, it reflects six major chronic health conditions that were consistently asked across NHANES waves and typically require regular doctor visits and management with medications. Twenty-three percent of respondents taking antipsychotics had not seen a mental health professional in the past 12 months, suggesting nearly one-quarter of antipsychotic prescriptions may be provided by non-psychiatrist physicians.

NHANES does not allow for distinction of whether a medication was prescribed for indicated or non-indicated uses, although slightly less than two thirds of respondents taking antipsychotics report schizophrenia, bipolar disorder, or major depressive disorder as a reason for use. From this information alone, we cannot confirm that these individuals are all taking antipsychotics for indicated uses, but findings are similar to previous studies demonstrating that off-label use of antipsychotics is a common clinical practice in adults [14]. The most common reason for off-label use of antipsychotics in our findings was insomnia or other sleep disorders, followed by a number of non-specific reasons relating to mental illness. Less common off-label reasons for use, including post-traumatic stress disorder, unspecified mood disorders, anxiety disorder, degenerative diseases of nervous system, panic disorder, obsessive-compulsive disorder, and pervasive developmental disorder were similar to previous studies in adults and Medicare patients [14, 15]. Even with intensive scrutiny to reduce off-label usage of antipsychotics for conditions other than the approved use, our study confirms the practice continues.

### Limitations

NHANES provides a useful description of the health care access, behaviors, and health status of a majority of U.S. adults, yet the data are not without limitations, as it does not sample some subpopulations that disproportionately have serious mental illness or take antipsychotic medications. The study design is cross-sectional, and as such, we are not able to observe the use of these medications in the sample over time, nor can we suggest causation in the association between antipsychotics and any of the covariates. Our study pools 6 years of NHANES data to obtain a more stable estimate of antipsychotic medication prevalence among adults during the time frame.

NHANES does not sample nursing home residents or incarcerated individuals, whose antipsychotic medication use has been shown to be substantially higher than the general population [16–18]. Individuals with serious mental illness are more likely to be homeless, and NHANES also does not sample homeless individuals. Therefore, our population estimates of antipsychotic use likely do not capture the entire demographic most likely to take antipsychotic medications [19]. Finally, although NHANES collects extensive interview and examination data, it does not allow for estimation of untreated or undiagnosed mental illness or psychosis.

## Conclusions

The current study is the first to use NHANES to estimate prescription antipsychotic use in U.S. adults. The study provides a useful replication of past outpatient data estimates, while also adding socio-demographic context on the use of antipsychotic medications in the general population [11]. The average U.S. adult taking a prescribed antipsychotic medication is taking more than 5 total prescription medications, two more than other adults. Higher prevalence of comorbid chronic diseases and poorer self-reported health in individuals taking antipsychotic medications reflect the need for careful management of mental health medications in conjunction with other chronic disease treatments. Antipsychotics are used for a variety of health conditions besides serious mental illness, and are often prescribed by non-psychiatrist physicians [6, 7]. With nearly 4 million U.S. adults taking antipsychotic medications, drug-drug interactions and adverse effects on comorbid conditions remain a relevant concern that merits continued monitoring and awareness.

## Data Availability

The datasets analyzed during the current study are publicly available for download from the National Center for Health Statistics at the Centers for Disease Control. https://www.cdc.gov/nchs/nhanes/index.htm

## Abbreviations

U.S.: United States
NHANES: National health and nutrition examination survey
OLS: Ordinary least squares
